# Dynamics of Orthopoxvirus Stability Under Simulated Luminal Conditions of the Gastrointestinal Tract for Assessing the Feasibility of Oral Immunization

**DOI:** 10.3390/vaccines14080681

**Published:** 2026-08-07

**Authors:** Lespek Kutumbetov, Moldir Azanbekova, Balzhan Myrzakhmetova, Moldir Tuyskanova, Aisulu Valieva, Nuraiym Sarsenkulova, Gulzhan Zhapparova, Dias Muzarap, Muratbay Mambetaliyev, Sanat Kilibayev, Kuandyk Zhugunissov

**Affiliations:** Research Institute for Biological Safety Problems LLP, Guardeyskiy 080409, Kazakhstan; l.kutumbetov@biosafety.kz (L.K.); m.serzhankyzy@biosafety.kz (M.T.); a.dulatbekovna@biosafety.kz (A.V.); n.sarsenkulova@biosafety.kz (N.S.); g.zhapparova@biosafety.kz (G.Z.); d.muzarap@biosafety.kz (D.M.); m.mambetalyev@biosafety.kz (M.M.); s.kilibayev@biosafety.kz (S.K.); k.zhugunisov@biosafety.kz (K.Z.)

**Keywords:** Monkeypox, orthopoxvirus, oral vaccine, gastrointestinal stability, enteric-coated capsules, vaccinia virus, immunization

## Abstract

Background/Objectives: Mpox (formerly monkeypox) is a re-emerging global health threat. While current vaccines are injectable, oral vaccination offers a painless alternative, though the harsh gastrointestinal (GI) environment challenges live viral vaccines. This study evaluated the stability of orthopoxviruses under simulated GI conditions to assess the physicochemical feasibility of oral mpox immunization. Methods: Attenuated and virulent strains of vaccinia, cowpox, and camelpox viruses were exposed to simulated gastric (pH 1.0–2.0 and 3.0–4.0 with pepsin) and intestinal (pH 7.0–7.5) conditions at 37 °C for 180 min. Viral titers were determined via cell culture assays. The physicochemical protective efficacy of enteric-coated capsules was evaluated using standard dissolution testing parameters. Results: All orthopoxviruses were rapidly inactivated under highly acidic fasting conditions (pH 1.0–2.0). However, they exhibited high stability at pH 3.0–4.0 and 7.0–7.5, retaining approximately 20–30% of their initial infectivity after 180 min. Enteric-coated capsules successfully maintained shell integrity in simulated gastric fluids for over 3 h and dissolved completely under intestinal conditions within ~130 min, aligning with small intestine transit times. Conclusions: Orthopoxviruses possess sufficient intestinal stability to support the physicochemical feasibility of oral immunization, provided they are protected from gastric acidity. Enteric-coated capsules represent a highly suitable delivery system for the intestinal release of live orthopoxvirus-based candidates, warranting further in vivo preclinical evaluation.

## 1. Introduction

Monkeypox is a serious infectious disease caused by the monkeypox virus (MPXV), a member of the genus Orthopoxvirus within the family Poxviridae. In addition to infecting non-human primates, the virus is pathogenic to humans, causing a disease clinically resembling smallpox [1]. Historically, monkeypox was reported predominantly in non-human primates, with only sporadic human cases documented in countries of the African continent [2]. Over the past decade, however, the disease has spread beyond Africa, and since 2022, large-scale outbreaks have been reported in Europe, Asia, and the Americas [3,4]. To date, more than 50,751 laboratory-confirmed human cases of monkeypox have been officially reported across 96 countries, including 206 fatalities [4]. Human-to-human transmission occurs primarily through close physical contact, prolonged face-to-face interaction, or sharing the same enclosed environment [5]. According to researchers led by Jude N. Williams at the Sierra Leone Institute for Disease Prevention and Control [6], the disease has recently reached epidemic proportions in Sierra Leone, with more than 3500 cases reported in 2025 alone. The infection predominantly affects young adults, children, and individuals living with human immunodeficiency virus (HIV) infection.

According to the World Health Organization (WHO) classification, the MPXV is categorized as a high-consequence pathogen (Risk Group 2) with pandemic potential [7]. Consequently, the WHO has recommended the development of effective countermeasures, including preventive and control strategies, to protect against this emerging threat to the human population [8].

In response to the WHO recommendations, the Ministry of Health of the Republic of Kazakhstan launched the targeted research program Development of Diagnostic Tools and Specific Prophylactic Measures against Monkeypox [9]. The program aims to develop diagnostic assays for the detection of the MPXV genome and MPXV-specific antibodies, as well as to develop both live attenuated and subunit vaccines against monkeypox.

MPXV is genetically and antigenically closely related to other members of the genus Orthopoxvirus, including cowpox virus, variola virus, camelpox virus, horsepox virus, vaccinia virus, and several other orthopoxviruses [1,10]. Consequently, attenuated strains of these viruses may serve as promising vaccine candidates against monkeypox, as has previously been demonstrated for cowpox virus [11] and vaccinia virus used for smallpox vaccination [10,12]. Vaccinia virus has also been shown to induce highly effective cross-protective immunity against camelpox [12].

Accordingly, vaccinia virus has been used by virtually all researchers as the genetic and antigenic backbone for the development of monkeypox vaccines. Examples include ACAM2000 (USA), JYNNEOS (Germany–Denmark), LC16m8 (Japan), OrthopoxVac (Russia), and other vaccine candidates [13,14,15,16]. This approach is based on the extensive historical use of vaccinia virus in smallpox vaccination, reflecting its close genetic and antigenic relationship with MPXV.

In the present study, attenuated strains of vaccinia virus, cowpox virus, and camelpox virus from the institute’s microorganism collection were selected as candidate platforms for the development of a monkeypox vaccine.

Currently available live monkeypox vaccines, including replication-competent, minimally replicating, and non-replicating formulations, are administered by scarification, subcutaneous injection, or intramuscular injection [13,14,15]. These routes of administration are associated with skin injury or needle puncture, patient discomfort and pain, as well as logistical challenges related to vaccine administration and dose delivery. In contrast, oral (per os) administration in the form of capsules or tablets containing a predefined individual dose offers a simple, convenient, and painless alternative that does not require specialized healthcare facilities or trained medical personnel.

Accordingly, the present study aimed to evaluate the stability of attenuated cowpox virus, vaccinia virus, and camelpox virus strains, selected as potential candidates for monkeypox vaccine development, and to determine an appropriate strategy for protecting these viruses under simulated gastrointestinal luminal conditions. The gastrointestinal environment was modeled with respect to hydrogen ion concentration (pH), temperature, and exposure time to assess the feasibility of oral immunization.

## 2. Materials and Methods

The study used attenuated vaccinia virus strains RIBSP-V and RIBSP-B, with infectious titers of 10^5.25^ and 10^5.50^ TCID_50_/mL, respectively; the attenuated cowpox virus strain CP-65K (10^5.50^ TCID_50_/mL); the virulent cowpox virus strain Cowpox-CAM (10^6.00^ TCID_50_/mL); and the virulent camelpox virus strain M-96 (10^4.50^ TCID_50_/mL). The viruses were tested both as cell culture suspensions and as lyophilized preparations.

Virus detection and infectivity titration were performed using primary and passaged lamb kidney (LK) cell cultures grown as monolayers in 96-well tissue culture plates. The cells were propagated in semisynthetic adherent growth medium (PSP) supplemented with 10% fetal bovine serum (FBS) and maintained in the same medium containing 2% FBS.

The Vero cell line used for viral propagation and titration was originally obtained from the American Type Culture Collection (ATCC, Manassas, VA, USA). The orthopoxvirus strains evaluated in this study included two attenuated strains of Vaccinia virus (VACV), a virulent and an attenuated strain of Cowpox virus (CPXV), and a virulent and an attenuated strain of Camelpox virus (CMLV). The Vero cell line and all viral strains were obtained from the State Collection of Microorganisms and the institutional biological repositories of the Research Institute for Biological Safety Problems (Gvardeyskiy, Kazakhstan), where they are authenticated, maintained, and quality-controlled in accordance with established institutional biosafety procedures.

Model solutions with different pH values were prepared using a 10% hydrochloric acid solution, which was prepared by dilution of concentrated 36% hydrochloric acid.

**Figure 1:**
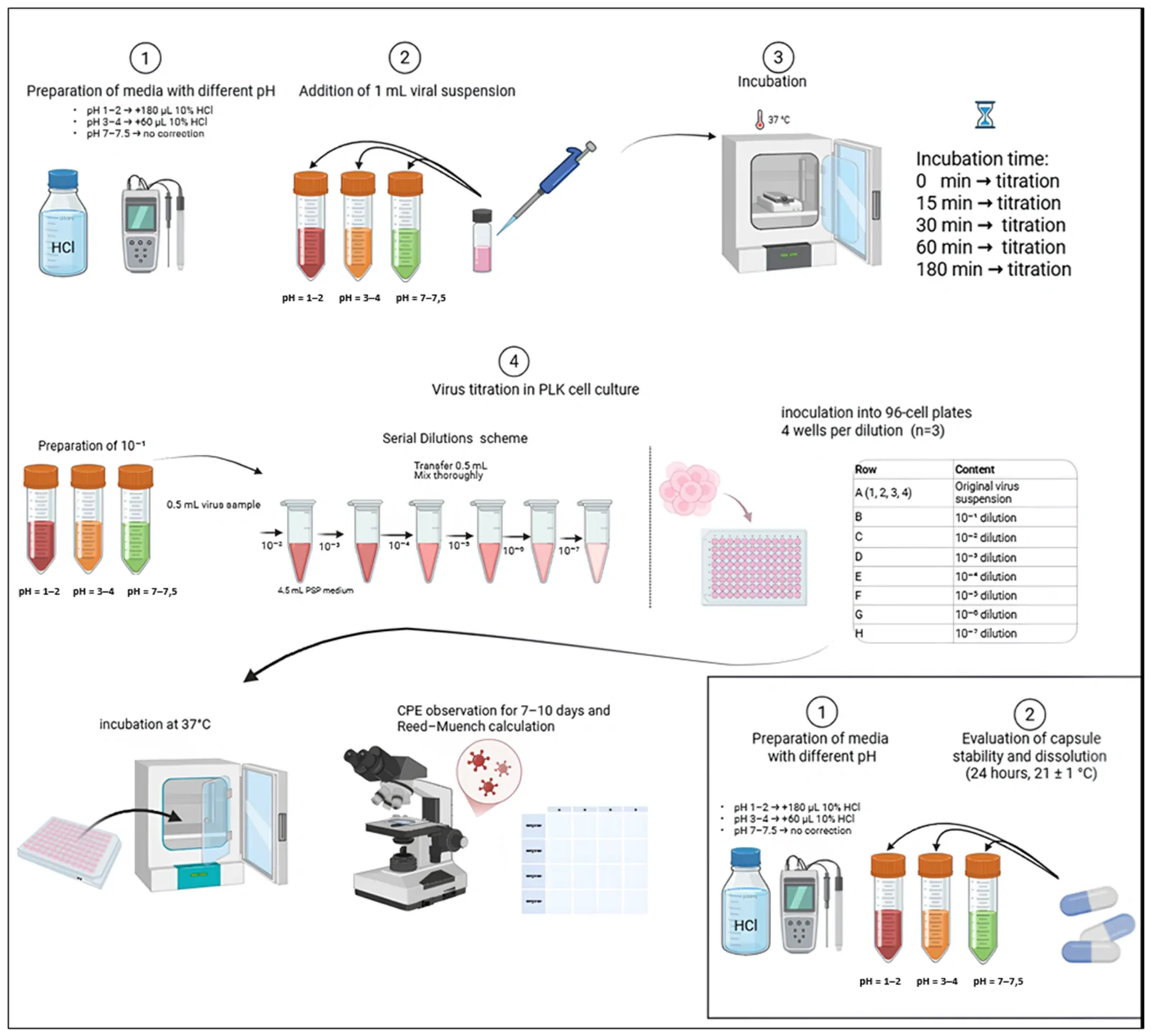
Experimental workflow for evaluation of viral stability at different pH conditions and capsule dissolution.

In several experiments, enteric-coated and gastric-soluble pharmaceutical capsules were employed to protect the viruses from external environmental factors. The study design is presented in Figure 1.

It is important to clarify that the capsule dissolution experiments were designed as physicochemical evaluations of the polymer shell’s integrity according to standard pharmacopeial guidelines (USP <711>), rather than direct virological assays. Direct measurement of viral infectivity inside an intact, undissolved capsule in acidic media is physically unfeasible without disrupting the shell. Therefore, the protective efficacy was evaluated indirectly by combining the in vitro stability data of the free virus with the physicochemical release kinetics of the enteric-coated capsules.

Preparation of a 10% Hydrochloric Acid Solution. A 10% hydrochloric acid solution was prepared by diluting concentrated 36% hydrochloric acid with sterile distilled water. The required volumes of the stock solution and sterile distilled water were calculated using Equation (1):

C_1_ × V_1_ = C_2_ × V_2_ (1)

where

C_1_—the concentration of the stock hydrochloric acid solution (36%);
V_1_—the volume of 36% hydrochloric acid required for preparation of the working solution;
C_2_—the target concentration (10%);
V_2_—the final volume of the solution (10 mL).

Substituting the corresponding values into Equation (1):

36 × V_1_ = 10 × 10

$$V_{1}=\frac{100}{36}\approx 2.78\;\mathrm{mL}$$

Since 100 mL of the concentrated hydrochloric acid solution contains 36 mL of HCl, the calculated amount of HCl required in 10 mL of a 10% solution was 2.78 mL. Therefore, 2.78 mL of the concentrated hydrochloric acid solution was diluted with sterile distilled water to a final volume of 10 mL to prepare the 10% hydrochloric acid solution.

Simulation of gastrointestinal tract conditions based on pH, temperature, and exposure time. Simulated gastrointestinal (GI) luminal conditions were established by reproducing the physiological pH values characteristic of different regions of the GI tract. Acidic conditions with pH values of 1.0–2.0 simulated the fasting stomach, which is characterized by a high concentration of hydrochloric acid and pronounced proteolytic activity. To evaluate viral stability under diverse physiological scenarios, pH values of 3.0–4.0 were utilized to simulate the postprandial (fed) stomach, where gastric acidity is partially buffered by food intake. This approach aligns with the core parameters of standard Fed-State Simulated Gastric Fluid (FeSSGF) without the introduction of complex organic components. This specific range was selected to model the maximum potential impact of buffered acidic conditions on viral replicative activity prior to intestinal transit. Neutral conditions with pH values of 7.0–7.5 simulated the small intestine, where acidic chyme is neutralized by bicarbonate-rich secretions and the luminal pH typically ranges from 6.5 to 7.5 or higher [17,18,19].

**Figure 2:**
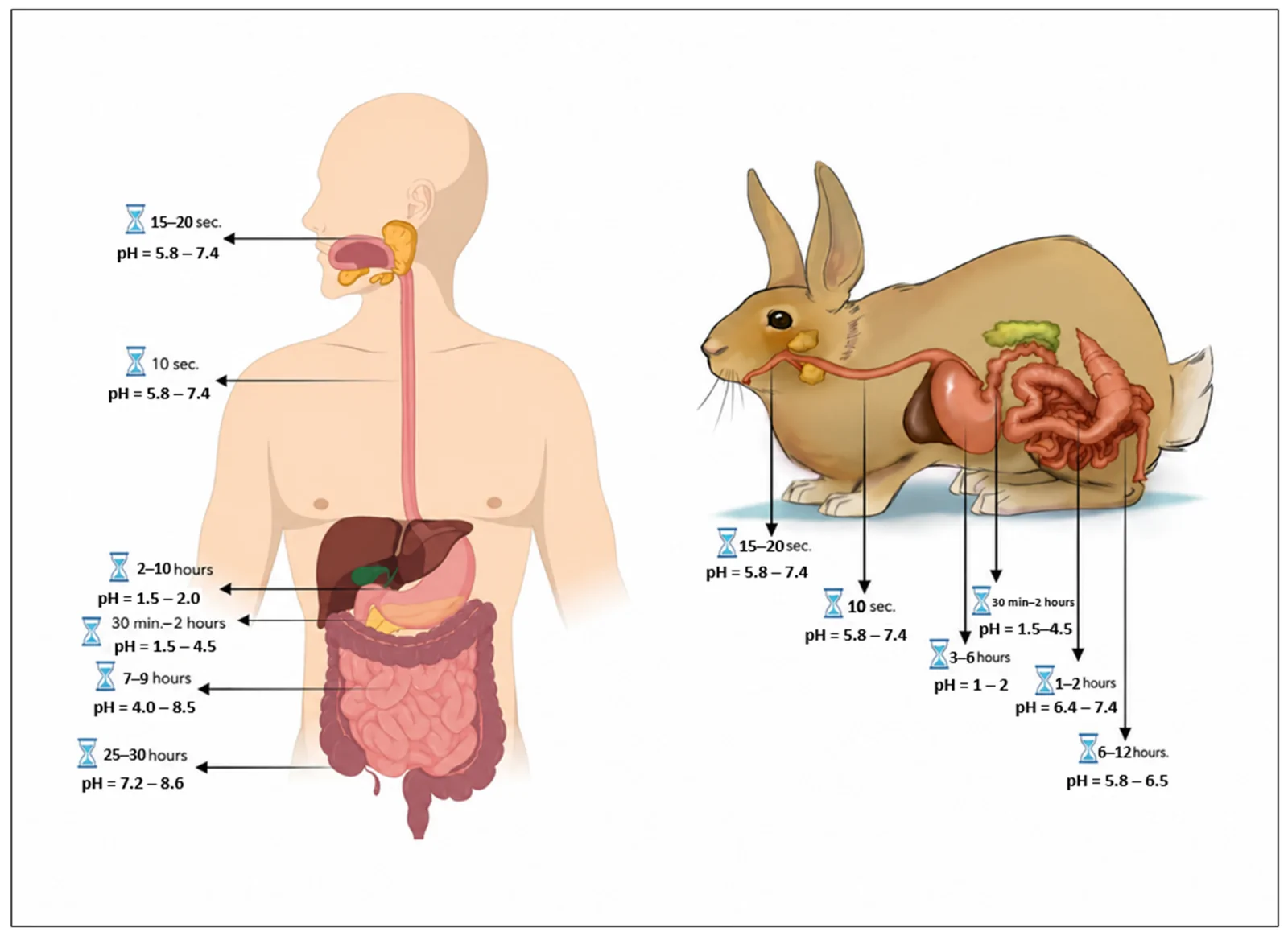
Physiological parameters of the gastrointestinal (GI) tract in humans and rabbits (a potential model for preclinical evaluation of orally administered formulations) [18,19].

The physiological temperature of the human and rabbit GI tract was simulated by incubating the samples at 37 °C. Exposure times ranging from 15 to 180 min were used to simulate the transit of the viral preparations through successive regions of the GI tract. A schematic representation of the anatomical and physiological organization of the human and rabbit GI tract is presented in Figure 2 [18,19].

Media with pH values of 1.0–2.0 and 3.0–4.0 were prepared using a 10% hydrochloric acid solution. Cell culture medium with its inherent pH was used to simulate conditions at pH 7.0–7.5. When necessary, the pH of the culture medium was adjusted using a sodium bicarbonate solution.

A 10% hydrochloric acid solution was prepared from 36% concentrated hydrochloric acid by diluting 2.78 mL of the stock solution with sterile distilled water to a final volume of 10 mL.

For each virus, 9 mL of cell culture medium was dispensed into three penicillin vials. In the first vial, the pH of the medium was adjusted to 1.0–2.0 by adding 180 μL of the 10% hydrochloric acid solution. In the second vial, the pH was adjusted to 3.0–4.0 by adding 60 μL of the 10% hydrochloric acid solution. The pH of the medium in the third vial was measured, and no adjustment was made if the pH was within the range of 7.0–7.5.

After adjustment of the desired pH, pepsin (Meito, Japan) was added to each vial at a final concentration of 2.0 mg/mL (18 mg per vial containing 9 mL of medium), followed by the addition of 1 mL of the viral suspension (10^−1^ dilution). An aliquot of the resulting virus suspension was collected immediately, without prior incubation, and titrated in LK cell culture to determine the baseline infectious titer. The remaining samples were incubated at 37 °C, corresponding to the physiological body temperature of humans and rabbits, for 15, 30, 60, and 180 min. The residual infection titer was determined after each incubation interval.

Determination of viral titers. Tenfold serial dilutions of each test sample were prepared in maintenance medium, and each dilution was inoculated into four wells of LK cell cultures in three independent replicates. Control wells received maintenance medium instead of the virus. The inoculated cultures were incubated at 37 °C for 7–10 days, after which viral infectivity was assessed by microscopic examination for the presence of a virus-induced cytopathic effect (CPE). Wells exhibiting CPE were considered positive, whereas those without CPE were considered negative. Viral titers were calculated using the Reed and Muench method [20].

Protection of viral preparations. To protect the viruses from the physicochemical conditions of the gastrointestinal (GI) tract, including acidic pH, 37 °C, and pepsin, the vaccine preparations were encapsulated. In one series of experiments, gastric-soluble capsules were used, whereas in another series, enteric-coated capsules were employed.

Evaluation of capsule stability and dissolution. To evaluate the stability of the capsule shells under conditions simulating different regions of the gastrointestinal (GI) tract, the capsules were immersed in solutions with different pH values containing pepsin and incubated at 37 °C. Their condition was monitored periodically for 24 h. Capsule stability was assessed visually based on the maintenance of shape, shell integrity, and sealing properties, as well as the presence of softening, deformation, or complete dissolution of the capsule shell. The time to loss of capsule integrity was recorded as the endpoint of capsule stability.

Statistical analysis. Statistical analyses were conducted using a two-way analysis of variance (two-way ANOVA) to evaluate the effects of both pH conditions and incubation time, followed by Tukey’s post hoc test for multiple comparisons. Differences were considered statistically significant at *p* < 0.05. All statistical analyses were carried out using GraphPad Prism (version 9.0).

## 3. Results

The results of infectious titer determination performed immediately after mixing the viruses with solutions of different pH values containing pepsin and after subsequent incubation at 37 °C demonstrated that the simulated conditions of different regions of the gastrointestinal (GI) tract had differential effects on the preservation of viral infectious titers.

**Figure 3:**
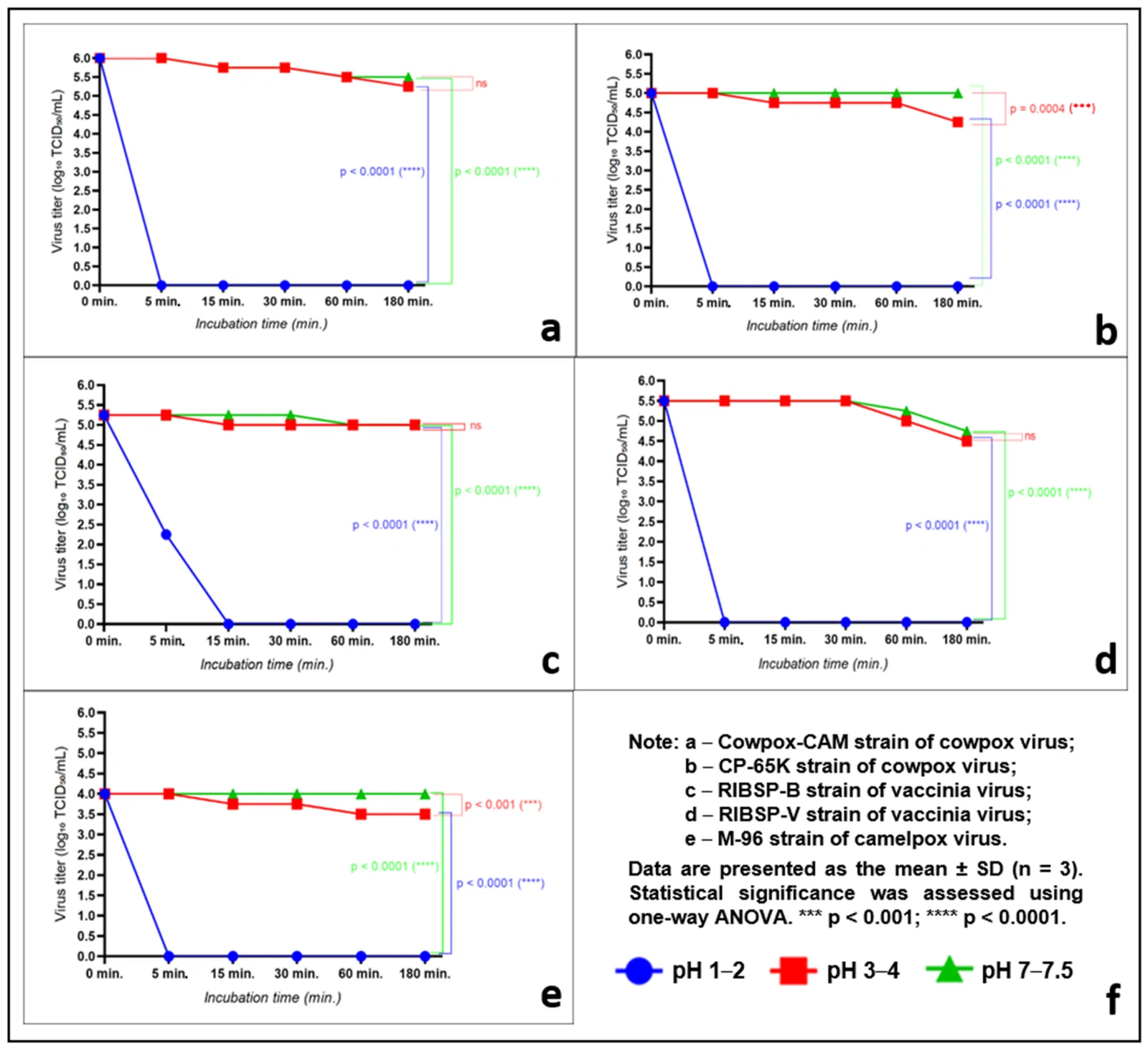
Effect of pH on the stability of Orthopoxvirus strains during incubation at 37 °C. The data is presented as an average value ± SD (*n* = 3). Statistical significance was assessed using a one-way analysis of variance. ns—not significant; **** *p* < 0.0001. (**a**) Effect of pH on the stability of the cowpox virus strain Cowpox-CAM during incubation at 37 °C. (**b**) Effect of pH on the stability of the cowpox virus strain CP-65K during incubation at 37 °C. (**c**) Effect of pH on the stability of the vaccinia virus strain RIBSP-B during incubation at 37 °C. (**d**) Effect of pH on the stability of the vaccinia virus strain RIBSP-V during incubation at 37 °C. (**e**) Effect of pH on the stability of the camelpox virus strain M-96 during incubation at 37 °C. (**f**) Notes.

The results obtained with the virulent cowpox virus strain Cowpox-CAM demonstrated that the preservation of its infectious titer was strongly dependent on pH under pepsin-containing conditions (Figure 3a).

Under pH 1.0–2.0 conditions in the presence of pepsin, the virus completely lost its virus titer immediately upon contact with the solution or within the few minutes (<5 min) required for transfer to the LK cell monolayer. At all subsequent sampling time points, no replication-competent virus was detected in samples maintained under these conditions. These findings indicate that cowpox virus is completely inactivated within the first few minutes after exposure to simulated gastric conditions and consequently loses its ability to infect host cells.

In the presence of pepsin at pH 3.0–4.0, the cowpox virus remained stable throughout the observation period. From an initial virus titer of 10^5.92 ± 0.08^ TCID_50_/mL, the titer decreased only slightly to 10^5.67 ± 0.08^ TCID_50_/mL after 15 and 30 min of incubation, to 10^5.42 ± 0.08^ TCID_50_/mL after 60 min, and to 10^5.17 ± 0.08^ TCID_50_/mL after 180 min. These results indicate that the cowpox virus retained its infectivity under simulated luminal conditions corresponding to the fed stomach and the gastroduodenal transition, with no significant inactivation observed during the 3 h observation period.

The virus exhibited the highest stability at pH 7.0–7.5. Under these conditions, the virus titer remained unchanged throughout the observation period at the initial level of 10^5.42 ± 0.08^ TCID_50_/mL, indicating that this environment had no appreciable effect on the infectivity of the virus.

Statistical analysis revealed significant differences between the results obtained under neutral conditions and those obtained at pH 1.0–2.0 (*p* < 0.0001), whereas the differences between the results obtained at pH 3.0–4.0 and pH 7.0–7.5 were not significant (ns).

Overall, these results demonstrate that the Cowpox-CAM strain of cowpox virus exhibits high stability over the pH range of 3.0–7.5, whereas it is rapidly and completely inactivated under highly acidic conditions at pH 1.0–2.0.

The results obtained with the attenuated cowpox virus strain CP-65K demonstrated, similarly to those observed in the first experiment, that the preservation of virus titer was strongly dependent on pH in the presence of pepsin (Figure 3b).

Under highly acidic conditions (pH 1.0–2.0), the attenuated cowpox virus, similarly to the virulent strain, completely lost its virus titer at the earliest stage of the experiment. No replication-competent virus was detected at any subsequent sampling time point, indicating complete viral inactivation under the simulated gastric conditions.

At pH 3.0–4.0, the attenuated cowpox virus retained its infectivity throughout the observation period. From an initial virus titer of 10^5.0 ± 0.14^ TCID_50_/mL, the titer decreased slightly to 10^4.83 ± 0.08^ TCID_50_/mL after 15, 30, and 60 min of incubation, and further declined to 10^4.25 ± 0.14^ TCID_50_/mL after 180 min. These findings indicate that viral inactivation under moderately acidic conditions was gradual and limited.

The highest virus stability was observed at pH 7.0–7.5. Throughout the incubation period at 37 °C, the virus titer remained stable at 10^5.00 ± 0.14^ TCID_50_/mL, with no evidence of a decline in infectivity. Statistical analysis revealed significant differences between the results obtained at pH 3.0–4.0 and pH 7.0–7.5 after 180 min of incubation (*p* = 0.0004). Likewise, the differences between the neutral conditions and those at pH 1.0–2.0 were highly significant (*p* < 0.0001). The neutral pH (7.0–7.5) group served as the positive thermal control, demonstrating the baseline thermal decay of the viruses at 37 °C in the absence of acidic or proteolytic stress.

Overall, these results demonstrate that the attenuated cowpox virus strain CP-65K exhibits high stability over the pH range of 3.0–7.5, whereas it is completely inactivated under highly acidic conditions (pH 1.0–2.0) in the presence of pepsin.

In subsequent experiments, the stability of the attenuated vaccinia virus strain RIBSP-B was evaluated under the same pH conditions as those used in the previous experiments (Figure 3c). The results demonstrated that this virus also remained sufficiently stable under moderately acidic, neutral, and mildly alkaline conditions.

Under highly acidic conditions (pH 1.0–2.0), the virus was rapidly inactivated. Immediately after inoculation into the medium, without incubation at 37 °C, the virus titer was 10^2.16 ± 0.08^ TCID_50_/mL. However, after 15 min of incubation, no detectable virus titer remained in the cell culture, and the virus remained undetectable throughout the entire observation period. These findings confirm the high sensitivity of the virus to extremely acidic conditions in the presence of pepsin.

At pH 3.0–4.0, the virus retained virtually its initial virus titer throughout the experiment. From an initial virus titer of 10^5.33 ± 0.08^ TCID_50_/mL, the titer decreased slightly to 10^5.00 ± 0.00^ TCID_50_/mL after 15 min of incubation and subsequently remained stable until 180 min. The plateau observed after the initial decline indicates that the virus was resistant to moderately acidic conditions. No statistically significant differences were detected between the results obtained at pH 3.0–4.0 and pH 7.0–7.5 (ns).

Under neutral conditions (pH 7.0–7.5), the virus also maintained a high virus titer. During the first 30 min, the virus titer remained at 10^5.25 ± 0.14^ TCID_50_/mL and then decreased slightly to 10^5.08 ± 0.08^ TCID_50_/mL after 60 and 180 min of incubation. The observed changes in virus titer indicate only minimal viral inactivation and confirm the high stability of the virus under neutral conditions. The differences between the neutral conditions and those at pH 1.0–2.0 were statistically significant (*p* < 0.0001).

Overall, these results demonstrate that the attenuated vaccinia virus strain RIBSP-B exhibits high stability over the pH range of 3.0–7.5, whereas it is rapidly and completely inactivated under highly acidic conditions (pH 1.0–2.0).

The second attenuated vaccinia virus strain, RIBSP-V, exhibited a pattern of pH stability similar to that observed for the other virus strains (Figure 3d).

Under highly acidic conditions (pH 1.0–2.0), the virus was completely inactivated at the earliest stage of the experiment. No replication-competent virus was detected throughout the incubation period, indicating complete viral inactivation under extremely acidic conditions.

At pH 3.0–4.0, the virus retained its initial virus titer of 10^5.42 ± 0.08^ TCID_50_/mL throughout the first 30 min of incubation. After 60 min, the virus titer gradually decreased to 10^4.92 ± 0.08^ TCID_50_/mL and further declined to 10^4.42 ± 0.08^ TCID_50_/mL after 180 min. The kinetics of the curve indicate an initial phase of stability followed by a moderate decline in virus titer, suggesting gradual viral inactivation under moderately acidic conditions.

Under neutral to mildly alkaline conditions (pH 7.0–7.5), the virus exhibited the highest stability. The initial virus titer of 10^5.42 ± 0.08^ TCID_50_/mL remained unchanged during the first 30 min of incubation. Thereafter, only a slight reduction in virus titer was observed, decreasing to 10^5.17 ± 0.08^ TCID_50_/mL after 60 min and to 10^4.67 ± 0.08^ TCID_50_/mL after 180 min. These findings indicate only limited viral inactivation under neutral conditions.

Statistical analysis revealed significant differences between the results obtained at pH 1.0–2.0 and pH 7.0–7.5 (*p* < 0.0001), whereas the differences between pH 3.0–4.0 and pH 7.0–7.5 were not statistically significant (ns).

Overall, these results demonstrate that the RIBSP-V strain of vaccinia virus exhibits high stability over the pH range of 3.0–7.5, whereas it is completely inactivated under highly acidic conditions (pH 1.0–2.0).

The virulent camelpox virus strain M-96 exhibited a pattern of virus titer preservation and decline across the tested pH conditions similar to that observed for the other virus strains (Figure 3e). Under highly acidic conditions (pH 1.0–2.0), the virus was completely inactivated at the earliest stage of the experiment. No replication-competent virus was detected throughout the remainder of the observation period, indicating complete viral inactivation under extremely acidic conditions.

At pH 3.0–4.0, the virus retained its virus titer throughout the observation period, although a gradual decline was observed. The initial virus titer was 10^3.92 ± 0.08^ TCID_50_/mL and decreased to 10^3.67 ± 0.08^ TCID_50_/mL after 15 and 30 min of incubation. With further incubation (60 and 180 min), the virus titer declined to 10^3.42 ± 0.08^ TCID_50_/mL. The kinetics of the curve demonstrate a gradual reduction in virus titer followed by stabilization after 30 min, indicating partial resistance of the virus to moderately acidic conditions.

For the M-96 strain at pH 3.0–4.0, the absolute reduction in infectivity was calculated as a 0.50 log_10_ TCID_50_/mL decrease (from $10^{3.92}$ to $10^{3.42}$) representing an absolute loss of approximately 68% of infectious particles over 180 min. This equates to an estimated survival of 2630 viable virions from an initial pool of 8318. The observed standard deviations (e.g., ±0.14) reflect the inherent biological and methodological variability of the cell-based Reed–Muench titration assay, which is entirely consistent with standard virological practices.

Under neutral conditions (pH 7.0–7.5), the virus exhibited greater stability. The initial virus titer was 10^3.92 ± 0.08^ TCID_50_/mL and remained unchanged throughout the subsequent incubation intervals at 10^3.92 ± 0.08^ TCID_50_/mL. The negligible change in virus titer indicates minimal loss of infectivity under neutral conditions.

Statistical analysis revealed significant differences between the results obtained at pH 1.0–2.0 and pH 7.0–7.5 (*p* < 0.0001), as well as between pH 3.0–4.0 and pH 7.0–7.5 after 180 min of incubation (*p* < 0.001).

Overall, these results demonstrate that the virulent camelpox virus strain M-96 retains its infectivity over the pH range of 3.0–7.5. However, compared with the other cowpox virus and vaccinia virus strains examined in this study, M-96 exhibited a greater reduction in virus titer during incubation.

A common finding for all virus strains examined was the complete loss of infectivity within a short period under highly acidic conditions (pH 1.0–2.0), simulating the fasting stomach. In all cases, the virus titer declined to undetectable levels at the earliest stages of incubation, indicating the high susceptibility of the orthopoxviruses tested to extremely low pH.

Under moderately acidic conditions (pH 3.0–4.0), all virus strains retained infectivity throughout the 180 min observation period, although a slow and gradual decline in virus titer was observed. Over this period, the reduction in virus titer was 10^0.75^ TCID_50_/mL for the virulent cowpox virus strain Cowpox-CAM, 10^0.50^ TCID_50_/mL for the virulent camelpox virus strain M-96, 10^0.50^ TCID_50_/mL for the attenuated cowpox virus strain CP-65K, and 10^0.50^–10^0.75^ TCID_50_/mL for the attenuated vaccinia virus strains RIBSP-B and RIBSP-V. These data indicate that, over the 180 min observation period under moderately acidic conditions simulating the luminal environment of the fed stomach and the gastroduodenal transition, the loss of viable virus particles ranged from approximately 70% to 80%. Thus, all virus strains exhibited comparable stability, with no substantial differences attributable to virus species or genetic and antigenic characteristics.

Under neutral conditions (pH 7.0–7.5), simulating the environment of the small intestine, all virus strains exhibited comparatively higher stability, and no significant differences in virus titer preservation were observed among the strains tested.

Overall, the results demonstrate that the orthopoxvirus strains examined possess sufficient stability over the pH range of 3.0–7.5. This stability may enable the preservation of viable virus particles during passage through the gastrointestinal tract, thereby supporting the feasibility of oral immunization.

Results of the capsule stability and dissolution study. The evaluation of capsule stability and dissolution under different pH conditions revealed distinct behaviors of gastric-soluble and enteric-coated capsules.

Under highly acidic conditions (pH 1.0–2.0), the enteric-coated capsules retained the integrity and hermeticity of their shells throughout the observation period, with no signs of dissolution. Similar stability was observed at pH 3.0–4.0, where the capsules also remained intact and did not dissolve during the entire observation period. Under neutral conditions (pH 7.0–7.5), simulating the environment of the small intestine, the enteric-coated capsules dissolved completely after 130 ± 2.05 min, accompanied by complete loss of shell integrity and hermeticity (Table 1).

Under highly acidic conditions (pH 1.0–2.0), the gastric-soluble capsules underwent gradual degradation and were completely transformed into a flocculent mass after 73 ± 5 min. At pH 3.0–4.0, complete capsule dissolution was not observed; however, marked swelling of the capsule shell developed during the 2 h incubation period. Similar changes were observed under neutral conditions (pH 7.0–7.5), where the capsules likewise did not dissolve but exhibited pronounced swelling throughout the 2 h incubation period (Table 2).

The results demonstrate that enteric-coated capsules are stable under acidic conditions and dissolve predominantly at neutral pH, whereas immediate-release capsules are less resistant to acidic environments and undergo pronounced swelling without complete dissolution at pH values above 3.0.

Overall, the findings indicate that the type of capsule shell has a substantial influence on its stability and dissolution behavior under different pH conditions. Enteric-coated capsules remained stable under acidic conditions simulating the gastric environment and dissolved completely under neutral conditions corresponding to those of the small intestine. These findings support their suitability as a delivery system for protecting the viral agent from gastric acidity and facilitating its release in the intestine.

In contrast, immediate-release capsules exhibited lower stability under highly acidic conditions, undergoing degradation or pronounced swelling without controlled dissolution at pH values above 3.0. Collectively, these results support the potential use of enteric-coated capsules as an appropriate dosage form for the intestinal delivery of immunobiological preparations based on the Orthopoxvirus strains evaluated in this study, thereby preserving viral viability during gastrointestinal transit and supporting the feasibility of oral immunization.

## 4. Discussion

The present study aimed to evaluate the stability of representative orthopoxviruses under conditions simulating the luminal environment of the gastrointestinal tract and to assess the feasibility of protecting these viruses for subsequent use in oral vaccines against MPXV.

The most important finding of this study was the demonstration that all viruses tested were inactivated almost instantaneously under highly acidic conditions (pH 1.0–2.0), simulating the fasting gastric environment. The pH-dependent inactivation profile of orthopoxviruses is a well-established biological phenomenon; indeed, foundational historical studies, such as those by Beard et al. (1938) [21], characterized the basic pH stability of vaccinia elementary bodies nearly a century ago. However, the scientific novelty of the present work lies in two critical modern contexts that extend far beyond these early baselines. First, this study evaluates specifically engineered, modern attenuated vaccine candidates (such as RIBSP-V and CP-65K), whose structural, biological, and genetic modifications may alter their environmental stability compared to historical wild-type strains. Second, the fundamental biological stability profiles of these modern strains were integrated for the first time with contemporary pharmaceutical enteric-coated delivery systems. While historical data provided the initial biological foundation, this study establishes the necessary contemporary physicochemical and pharmacokinetic framework required to engineer a viable, translationally relevant oral vaccine formulation. Regardless of virus species, virulence, or degree of attenuation, replication-competent virus was no longer detectable within the first few minutes of exposure to the acidic medium. These findings indicate that extremely low pH is the principal limiting factor preventing the survival of orthopoxviruses during gastric transit. A similarly high sensitivity of viral particles to highly acidic conditions has been reported for a variety of viruses, including members of the Poxviridae family, and has been attributed to the denaturation of viral proteins and disruption of virion integrity [22,23,24].

It should be emphasized that, in the present study, the effects of acidity were evaluated in combination with pepsin, the principal proteolytic enzyme of gastric juice, rather than in isolation. Pepsin is known to exhibit maximal enzymatic activity at pH 1.5–2.5, where it catalyzes the hydrolysis of protein structures in biological substrates [17,25]. The outer membrane and surface proteins of orthopoxviruses play a critical role in host cell attachment and the initiation of infection [26,27]. Therefore, the combined effects of a high hydrogen ion concentration and the proteolytic activity of pepsin are likely to promote rapid denaturation of viral proteins, disruption of virion structure, and complete loss of viral infectivity [17,25].

An equally important finding was that all viruses examined retained infectivity in the presence of pepsin at pH 3.0–4.0. Despite the presence of the enzyme, virus titers were maintained throughout the entire 180 min observation period, with reductions not exceeding 0.50–0.75 log_10_ TCID_50_/mL. These findings indicate that increasing the pH to 3.0–4.0 markedly reduces the detrimental effects of the gastric environment on viral particles, even in the presence of pepsin. This observation is likely explained by the sharp decline in pepsin activity as pH increases [28]. The present findings are consistent with previous reports demonstrating that viral stability depends on the acid–base properties of the surrounding environment and that changes in hydrogen ion concentration alter the conformational organization of viral proteins [17,22,25].

Particular attention should be given to the absence of statistically significant differences in stability among the orthopoxvirus strains examined. Despite differences in origin, virulence, and degree of attenuation, all viruses exhibited similar patterns of virus titer preservation and decline under identical pH conditions. These findings suggest that members of the genus Orthopoxvirus share common structural and functional mechanisms governing their resistance to gastrointestinal environmental factors [17,29,30,31].

Under neutral conditions (pH 7.0–7.5), simulating the environment of the duodenum and small intestine, all viruses retained high virus titers throughout the observation period. Under these conditions, the effects of gastric acidity and pepsin were essentially absent, and only minimal reductions in virus titer were observed. These findings are consistent with previous reports demonstrating the high stability of vaccinia virus and other orthopoxviruses under physiological conditions [24,32,33].

The present findings have important practical implications. The principal challenge in the development of a live oral vaccine based on orthopoxviruses is not the stability of the virus within the intestine, but rather its protection against the detrimental effects of the gastric environment. Once the gastric barrier is overcome, the viruses evaluated in this study retain a substantial proportion of their infectivity for at least 3 h, potentially allowing interaction with the intestinal mucosa and the gut-associated lymphoid tissue.

The results of the capsule evaluation further support this conclusion. Enteric-coated capsules exhibited high stability under acidic conditions (pH 1.0–4.0), maintaining shell integrity and hermeticity throughout the observation period. In contrast, they dissolved completely under neutral conditions, thereby releasing their contents. Immediate-release capsules, on the other hand, underwent degradation or pronounced swelling before reaching conditions representative of the intestine. These findings indicate that enteric-coated capsules can effectively protect the viral preparation from the combined effects of hydrochloric acid and pepsin in the stomach and facilitate its delivery to the intestine while minimizing the loss of viral infectivity. Similar gastro-resistant delivery systems are widely used in the development of oral pharmaceutical formulations and vaccines to protect bioactive substances from degradation in the gastric environment [21,34,35].

It should be acknowledged that the simulated gastrointestinal environment used in this study was intentionally simplified to isolate the primary barrier to oral viral delivery: gastric acidity. Specifically, a pH-adjusted cell culture medium supplemented with pepsin was used to simulate gastric conditions rather than pharmacopeial standard fluids (e.g., United States Pharmacopeia [USP]-compliant formulations) containing pancreatin and bile salts. The primary objective was to evaluate the immediate effects of gastric acidity and proteolytic enzymes, which, as demonstrated by our results, constitute the principal barrier to viral survival following oral administration. Although the omission of bile salts and standard gastrointestinal fluids represents a limitation for comprehensive simulation of the gastrointestinal environment, this experimental design was specifically intended to model the initial gastric transit stage. Notably, orthopoxviruses possess a structurally robust, multilayered virion architecture that has been reported to exhibit greater resistance to detergent- and bile-mediated disruption than many other enveloped viruses [36,37]. Therefore, overcoming the gastric environment represents the primary challenge for successful oral delivery of these viral candidates. Future in vivo preclinical studies, currently underway in rabbit models, will evaluate both viral survival and immunogenicity under physiological gastrointestinal conditions, inherently accounting for the full complexity of the intestinal environment.

An important pharmacokinetic consideration is the alignment between capsule dissolution time and gastrointestinal transit. The enteric-coated capsules dissolved completely in approximately 130 min under neutral conditions. Physiologically, this indicates that the viral payload would be released in the distal duodenum or proximal jejunum. Given that the total small intestinal transit time in humans ranges from 3 to 5 h [38], the released virions will have a sufficient temporal window (approximately 2 to 3.5 h) to transit through the jejunum and reach the Peyer’s patches in the terminal ileum—the primary inductive sites for gut-associated lymphoid tissue (GALT) immunity. This temporal alignment strongly supports the suitability of this delivery system.

A potential immunological risk of oral administration is the induction of mucosal tolerance [39]. While oral tolerance is a well-documented phenomenon for soluble protein antigens, it is largely bypassed by live, replicating viral vectors [40]. Replication of orthopoxviruses within the mucosal epithelium and local antigen-presenting cells provides strong ‘Danger’ signals (PAMPs/DAMPs) that override these tolerogenic pathways [40]. This promotes robust mucosal IgA and systemic IgG responses, as evidenced by the successful oral use of live viral vaccines, such as the oral polio vaccine (OPV) [41] and recombinant vaccinia-rabies vectors [42].

Furthermore, the potential advantage of an oral orthopoxvirus vaccine extends beyond systemic immunity to the induction of robust mucosal immunity. Human-to-human MPXV transmission is heavily driven by mucosal contact (oral, genital, rectal) [43]. An oral live vaccine could establish localized mucosal immunity at these primary portals of entry. The feasibility of this approach is strongly supported by the global success of the VACV-based rabies vaccine (RABORAL V-RG) [44], which is administered orally to wildlife via baits. In that model, the buccal and intestinal mucosa serve as the primary sites of viral entry and immune induction, proving that live orthopoxviruses can successfully initiate immunization via the oral route in real-world conditions.

In summary, the present study demonstrates that all orthopoxvirus strains evaluated exhibit comparable stability under simulated gastrointestinal conditions. Viral infectivity was completely lost under fasting gastric conditions as a result of exposure to the combined effects of gastric acidity and pepsin, whereas it was largely preserved under moderately acidic conditions and in the intestinal environment. These findings provide experimental support for the development of live oral vaccines against MPXV based on orthopoxviruses, provided that effective gastro-resistant delivery systems, particularly enteric-coated capsules, are used to protect the viral preparation during gastric transit [21,34,35,45].

## 5. Conclusions

Under in vitro conditions simulating the luminal environment of the gastrointestinal tract, all six attenuated and virulent strains of cowpox virus, camelpox virus, and vaccinia virus exhibited comparable patterns of virus titer preservation and decline. Under conditions simulating the fasting stomach (pH 1.0–2.0), all viruses were inactivated almost immediately. In contrast, under conditions simulating the fed stomach (pH 3.0–4.0), the inactivating effects of hydrochloric acid and pepsin were markedly reduced. Under conditions corresponding to the duodenum and subsequent regions of the intestine (pH ≥ 5.0–6.0), the denaturing effects of the acidic pepsin-containing environment were no longer observed.

All orthopoxvirus strains retained infectivity for at least 3 h under conditions simulating the fed stomach, the gastroduodenal transition, and the intestinal lumen. At the end of the observation period, the proportion of replication-competent virions remaining was approximately 20–30% of the initial level. These findings indicate that, if protected from the detrimental effects of the gastric environment and successfully delivered to the intestine, orthopoxviruses retain sufficient infectivity to support interaction with the intestinal mucosa for at least 3 h. Consequently, enteric-coated capsules represent a highly suitable delivery system to protect these live viral candidates from the acidic and enzymatic luminal environment of the stomach. During our testing, these capsules remained intact and hermetically sealed under simulated gastric conditions for at least 3 h—a period sufficient for gastric transit—and subsequently dissolved within approximately 2 h under intestinal conditions. The observed stability of these viruses under intestinal conditions, combined with reliable gastro-resistant encapsulation, provides experimental support for the further development of oral vaccine formulations based on orthopoxviruses, warranting further in vivo preclinical evaluation.

## Figures and Tables

**Table 1:** Stability of enteric-coated capsules under different pH conditions.

Exposure Time (min)	pH 1.0–2.0	pH 3.0–4.0	pH 7.0–7.5
0	Intact	Intact	Intact
60	Intact	Intact	Intact
120	Intact	Intact	Intact
130 ± 2.1	Intact	Intact	Dissolved (+)
180	Intact	Intact	Dissolved (+)

*Note*. “Intact” indicates maintenance of capsule shape, shell integrity, and hermeticity. “(+)” indicates complete loss of capsule hermeticity and dissolution. Data represent the consensus of *n* = 3 replicates.

**Table 2:** Stability of gastric-soluble capsules under different pH conditions.

Exposure Time (min)	pH 1.0–2.0	pH 3.0–4.0	pH 7.0–7.5
0	Intact	Intact	Intact
73 ± 5.0	Dissolved (+)	Swollen (±)	Swollen (±)
120	Dissolved (+)	Dissolved (+)	Dissolved (+)

*Note*. “(+)” indicates complete degradation/dissolution. “(±)” indicates pronounced swelling without complete dissolution. Data represent the consensus of *n* = 3 replicates.

## Data Availability

The authors declare that the data supporting the findings of this study are available within the article. The raw data are provided with this article.

## Abbreviations

The following abbreviations are used in this manuscript:

GI	Gastrointestinal
MPXV	monkeypox virus
HIV	human immunodeficiency virus
WHO	World Health Organization
TCID	Tissue Culture Infectious Dose
LK	lamb kidney
FBS	fetal bovine serum
CPE	cytopathic effect
